# 3D printed personalized wound dressings using a hydrophobic deep eutectic solvent (HDES)-formulated emulgel

**DOI:** 10.1039/d4ra05456c

**Published:** 2024-10-28

**Authors:** Gorawit Yusakul, Juntratip Jomrit, Rommel G. Bacabac, Aruna Prasopthum

**Affiliations:** a School of Pharmacy, Walailak University Thasala Nakhon Si Thammarat 80160 Thailand aruna.pr@mail.wu.ac.th; b Biomass and Oil Palm Center of Excellence, Walailak University Thasala Nakhon Si Thammarat 80160 Thailand; c Medical Biophysics Group, Department of Physics, University of San Carlos Nasipit, Talamban Cebu City 6000 Philippines

## Abstract

Curcuminoids, known for their antibacterial, anti-inflammatory, and wound healing properties, face challenges in medical applications due to their limited water solubility, resulting in poor bioavailability and clinical efficacy. This study introduces a novel approach to formulating 3D printing ink for personalized wound dressings by utilizing hydrophobic deep eutectic solvents (HDES) to incorporate poorly water-soluble compounds from *Curcuma longa* (*i.e.*, curcuminoids and ar-turmerone) into hydrogels. The use of HDES, comprising either acetic acid or octanoic acid combined with menthol in a 2 : 1 molar ratio, significantly improved the solubility of curcuminoid derivatives and ar-turmerone by approximately 10 to 600 times, depending on the intrinsic chemical polarities of each compound, compared to conventional extraction solvents (*i.e.*, ethanol and water). By formulating an emulgel using HDES as the oil phase in a gelatin methacryloyl (GelMA) solution stabilized by a biocompatible surfactant, we achieved a 3D biocompatible printing ink with preserved rheological characteristics, enabling the production of personalized wound dressings using a custom-designed, syringe-based 3D printer. The emulgel constructs exhibited regulated swelling profiles, prolonged release of curcuminoids over 60 days as monitored by a Franz cell diffusion assay, and promoted human dermal fibroblast proliferation *in vitro*. Additionally, the emulgel components worked synergistically with curcuminoids to significantly enhance anti-biofilm activity against *Staphylococcus aureus*, offering an effective strategy to prevent wound infections. Our findings have demonstrated, for the first time, the formulation of biochemical ink for 3D printing harnessing HDES, providing a new pathway for developing advanced wound dressings with relatively high concentrations of poorly soluble plant bioactive compounds tailored for chronic wound management.

## Abstract 2

Menthol/fatty acid HDES enables higher incorporation of curcuminoids in water-rich matrices (3D printed hydrogel). It promotes fibroblast proliferation and, with curcuminoids, synergistically improves anti-biofilm activity.

## Graphical abstract

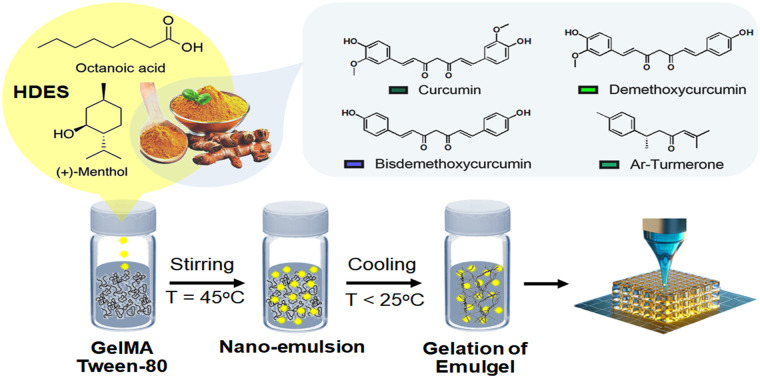

## 1. Introduction

Chronic wounds, such as diabetic foot ulcers, frequently exceed the typical healing timeline, often remaining unresolved for weeks or months and posing a substantial challenge to healthcare management.^1^ These wounds are particularly problematic in diabetic patients, where the condition's hyperglycemic state disrupts the orderly sequence of wound healing—comprising hemostasis, inflammation, proliferation, and remodeling.^1,2^ Ideally, these phases work in harmony to stop bleeding, eliminate pathogens, foster tissue regeneration, and rebuild the wound area with new extracellular matrix proteins.^3^ However, diabetes-induced chronic wounds exhibit a prolonged inflammatory response while the formation of healing tissue is significantly hindered.^2^ Moreover, the extended exposure of such wounds creates a conducive environment for a wide array of bacteria both pathogenic and opportunistic including *Pseudomonas aeruginosa* (*P. aeruginosa*), *Staphylococcus aureus* (*S. aureus*), and *Escherichia coli* (*E. coli*) to proliferate and form biofilms,^4^ increasing the risk of infection and complicating the healing process. This impaired healing trajectory underscores the need for innovative approaches to manage chronic wounds effectively, especially in patients with underlying conditions like diabetes.

Recent decades have seen remarkable advancements in wound dressing technologies, with an emphasis on designs that closely replicate the skin's complex architecture and microenvironment.^5,6^ Ideal wound dressings offer moisture balance, antimicrobial activity, oxygen permeability, and support for tissue regeneration.^7^ A moist environment is crucial for stimulating fibroblast and keratinocyte proliferation, leading to collagen and tissue neoformation.^8^ Incorporating antimicrobial agents into these therapies extends their efficacy and release over the healing duration.^9^ The integration of 3D printing technology represents a significant leap forward, allowing for the intricate wound dressing fabrication resembling human skin structures.^10^ Fabrication of wound dressings by 3D printing also facilitates the blending of natural and synthetic materials, alongside growth factors, drugs, and cells, enhancing the dressings' biocompatibility, bioactivity, and mechanical robustness.^11^ The precise control over drug release profiles achievable by adjusting the infill density in 3D printed structures permits personalized treatments tailored to individual patient needs. Hydrogels, owing to their capacity to sustain a moist wound environment and allow oxygen and metabolite transfer, are considered optimal for wound dressing fabrication by 3D printing.^10,11^ Despite the advantages of hydrogels in maintaining a moist wound environment, their low mechanical strength prompts the exploration of more robust alternatives.

Gelatin methacryloyl (GelMA), derived from the chemical modification of gelatin,^12^ retains the essential properties of its precursor, including the presence of RGD motifs and MMP sequences, vital for cellular interactions and scaffold bioactivity.^13^ Unlike pure gelatin, which suffers from rapid degradation and low mechanical strength, GelMA offers improved mechanical properties, biocompatibility, and controlled biodegradability, making it an ideal candidate for wound healing applications. The photocrosslinkable nature of GelMA through both visible and UV light enables the fabrication of stable, structurally intact bioinks, overcoming the limitations of low-concentration hydrogels.^14^

*Curcuma longa* L. (turmeric) has long been renowned for its varieties of pharmacological properties, including antioxidant,^8^ anti-inflammatory,^15^ bactericidal,^16^ and wound-healing effects,^17^ primarily due to containing high concentrations of essential oils and curcuminoids. Curcumin and its derivatives such as bisdemethoxycurcumin and demethoxycurcumin, constitute 12–14% of the dry weight of *C. longa*.^18^ Notably, curcumin is recognized for its safety, even at doses up to 12 grams per day, and has received FDA approval for its therapeutic use.^19^ Additionally, essential oils from turmeric, including aromatic-turmerone have shown antioxidant and anti-inflammatory effects.^15^ The synergism between curcumin and turmeric oils enhances curcumin's anti-inflammatory actions, suggesting that their combined extraction could offer superior health advantages.^20^ However, the direct inclusion of these compounds in GelMA for 3D printed wound dressings poses challenges due to their low solubility and stability at physiological conditions. Previous efforts to incorporate curcumin into GelMA hydrogels have encountered challenges, such as direct dissolution in DMSO, which is limited by DMSO's cytotoxic effects, restricting the amount of curcumin that can be incorporated.^8^ Another approach utilized synthetic nanocarriers to encapsulate curcumin within nanoparticles for hydrogel compatibility.^21^ However, this method involves time-consuming additional synthetic steps to produce curcumin-loaded nanoparticles.

In this work, we have utilized deep eutectic solvents (DES) as an eco-friendly solvent for the effective extraction of curcuminoids and ar-turmerone from *C. longa*. Subsequently, we developed an emulgel formulation by blending DES directly with GelMA, establishing a novel medium that merges the characteristics of emulsions and gels.^22^ Hydrophobic DES (HDES) was chosen for fabricating 3D-printed personalized wound dressings due to its relatively superior solubility for curcuminoids and ar-turmerone compared to hydrophilic DESs as well as its ability to support a stable emulgel formulation.^23,24^ Capitalizing on the environmental advantages, biodegradability, and the remarkable chemical and thermal stability of these HDES,^24^ and building upon existing studies that demonstrate the potential of menthol-based HDES to enhance skin penetration^25^ and inherent antimicrobial properties of acetic acid^26^ and octanoic acid,^27^ which could be beneficial for wound healing, we chose a binary mix of acetic or octanoic acid with menthol in a 2 : 1 molar ratio. This combination has effectively dissolved curcuminoids and ar-turmerones, positioning it as an optimal solvent system within the hydrogel matrix for 3D printing applications. The resulting HDES-containing emulgel not only exhibited superior solubilization of curcuminoids and ar-turmerones but also showed the proper viscosity and gelation for 3D printing. Utilizing our custom-made syringe-based desktop 3D printer, we fabricated personalized wound dressings from this emulgel, demonstrating their sustained curcuminoid release, biocompatibility with human dermal fibroblasts, ability to promote cell proliferation, and efficacy in inhibiting biofilm formation by *S. aureus*. This pioneering approach of creating patient-specific wound dressings *via* 3D printing with HDES-containing emulgel has highlighted the innovative integration of natural compound solubilization with advanced fabrication techniques for enhanced wound care solutions.

## 2. Materials & methods

### 2.1 Materials

Acetic acid (99.8% purity) and acetonitrile (AR grade) were purchased from RCI Labscan Limited (Bangkok, Thailand). Curcumin (CUR, ≥99.5% purity), demethoxycurcumin (DEM, ≥98% purity), bisdemethoxycurcumin (BIS, ≥98% purity), gelatin type A, phosphate buffer saline (PBS, 0.01 M, pH 7.5), methacrylic anhydride (MA), hexamethyldisilane, and lithium phenyl-2,4,6-trimethylbenzoylphosphinate (LAP) were supplied by Sigma-Aldrich (Missouri, USA). Octanoic acid (≥98% purity) was provided by Tokyo Chemical Industry Co., Ltd (Tokyo, Japan), while aromatic-turmerone (≥90% purity) was obtained from Toronto Research Chemicals, Inc. (Ontario, Canada). Dulbecco's Modified Eagle Medium (DMEM), antibiotic/antimycotic Solution, fetal bovine serum (FBS), Quant-iT™ PicoGreen® assay kit, and LIVE/DEAD cell viability assay were obtained from ThermoFisher Scientific, UK. The Strat-M® membrane was sourced from Merck Millipore, USA, while SYTO™ 9 and propidium iodide were obtained from Invitrogen, UK. Tryptone Soya Broth (TSB) medium was purchased from HiMedia Laboratories Pvt. Ltd (Mumbai, India).

### 2.2 Formulation and characterization of HDES

HDESs were synthesized following a previously established protocol.^24^ In brief, two mixtures were prepared, one consisting of acetic acid and l-menthol in a molar ratio of 2 : 1, denoted as AAM-HDES, and the other comprising octanoic acid and l-menthol in the same molar ratio, referred to as OAM-HDES. This mixture was stirred at 45 °C for 20 minutes until a clear eutectic solution formed. After cooling to room temperature, the solutions were stored under ambient conditions. To verify their eutectic nature by thermal behavior, both HDESs and their individual components were analyzed using differential scanning calorimetry (DSC) using a DSC 6000 instrument equipped with an IntraCooler system (PerkinElmer, USA). During the DSC procedure, a sample of 12 mg was placed in aluminum DSC pans and sealed. The samples were initially equilibrated at 40 °C, then cooled to −50 °C and held for 3 minutes before being heated to 250 °C at a rate of 5°C min^−1^.

### 2.3 Solubility test of HDES

The solubility of bisdemethoxycurcumin, demethoxycurcumin, curcumin, and aromatic-turmerone (ar-turmerone) in HDESs was evaluated by using a *C. longa* ethanolic extract. This extract was prepared by macerating *C. longa* rhizomes in 95% (v/v) ethanol at 25 °C for 48 hours, then concentrated under vacuum and freeze-dried to remove moisture. For solubility tests, the ethanolic extract was added to 1 mL of HDES or their individual acid component in comparison until saturation, indicated by the presence of undissolved solids, and agitated at 37 °C for 72 hours. To obtain the solubility of the compounds within the conventional solvents, the extract was added to 1 mL 95% (v/v) ethanol/water or 1 mL distilled water. Subsequent centrifugation at 7155*g* for 10 minutes at 25 °C yielded clear solutions, which were subjected to High-Performance Liquid Chromatography with Diode-Array Detection (HPLC-DAD), employing a VertiSepTM USP C18 column (4.6 mm × 250 mm), 5 μm particle size; Vertical Chromatography Co. Ltd (Nonthaburi, Thailand), following the methodology outlined in our prior research,^24^ to measure the amount of each curcuminoid derivative and ar-tumerone.

### 2.4 Synthesis of gelatin methacrylol (GelMA) hydrogel

GelMA synthesis followed a previously published protocol,^28^ starting with dissolving skin porcine gelatin type A in PBS at 50 °C until a 10% w/v homogenous solution was obtained. Next, 8 mL of MA was added dropwise to the gelatin solution under stirring, and the reaction was allowed to proceed at 50 °C for 3 hours. The mixture was then 5-fold diluted with PBS at the same temperature to stop the reaction. For purification, the solution underwent dialysis against distilled water at 40 °C for 1 week, with water changes every 12 hours, using dialysis membranes with an 80 kDa molecular cutoff. The purified product was freeze-dried for 1 week, resulting in dry GelMA, which was stored at −80 °C until further use.

### 2.5 Formulation and characterization of emulgel

Both AAM- and OAM-HDESs, effectively extracting curcuminoids from turmeric, were integrated into a 10% (w/v) GelMA solution heated to 45 °C with 1% or 2% (v/v) Tween-80. This addition of HDES was performed dropwise with a flow rate of 0.1 mL min^−1^, controlled by a syringe pump (NE-300, New Era Pump Systems, Inc., USA) while stirring at 3000 rpm. Subsequently, the mixtures were homogenized at 13 000 rpm for 10 minutes and allowed to rest for 30 minutes at 37 °C in a temperature-controlled water bath to observe emulsion miscibility. The formed microemulsions were analyzed for particle size using a Zetasizer (Malvern ZETASIZER NANO-ZS ZEN 3600). Rheological properties of the emulsions, with various OAM- HDES concentrations in 10% (w/v) gelatin and 2% (v/v) Tween-80, were assessed using a HAAKE MARS rheometer (Thermo Scientific, UK) with a 25 mm diameter parallel plate at a 0.5 mm gap, measuring at 25 °C and a shear rate of 100 s^−1^. Gelation behavior was evaluated by measuring viscosity during a temperature decrease from 35 to 10 °C at a rate of 5°C min^−1^. Crosslinking durations for GelMA, both with and without 2% (v/v) Tween-80 and either AAM- or OAM-HDES, were established by exposing 1 mL of 12% (v/v) HDES in 10% w/v GelMA containing 2% (v/v) Tween-80 to visible (LED, 3.5 W, 550 Lm) light with 60 cm distance from the light source for intervals between 15 to 120 s. The complex modulus was then measured at a 1 Hz frequency following crosslinking. The procedures were performed in triplicate for three samples.

### 2.6 3D printing of emulgels

The microemulsion ink, comprising 10% (w/v) GelMA, 0.5% (w/v) lithium phenyl-2,4,6-trimethylbenzoylphosphinate (LAP), 2% (v/v) Tween-80, and 12% (v/v) OAM-HDES containing *C. longa* extract, was loaded into a 20 mL Nipro® plastic syringe, pre-warmed to 37 °C and then cooled to 4 °C for 10 minutes. This syringe, equipped with a 22G smooth flow tapered tip (Adhesive Dispensing Ltd, UK), was mounted onto the printhead of our custom-designed desktop 3D bioprinter. The bioprinter, specifically tailored for a 20 mL Nipro® syringe, features precise flow rate control (0.05 mL min^−1^) through a step motor with an 8 mm diameter shaft and a temperature regulation system for the polymer solution, adjustable from 4–80 °C. Constructed with a closed-box frame to isolate the printhead from external conditions, its internal wiring is neatly organized in cable trays, and the print base included clamps for securing a 6-well microwell plate. Prior to printing, the syringe was set to 25 °C for 10 minutes, optimizing the ink temperature. The wound dressing, a 10 × 10 mm^2^ square with a 50% or 80% infill connected monotonic pattern, was designed using Onshape and programmed with SuperSlicer. Post-printing, the construct was exposed to visible light for 30 seconds to fully crosslink the GelMA, thus finalizing the dressing's structure for its intended application.

### 2.7 Photostability test of curcuminoids and ar-turmerone in emulgels

To determine the remaining curcuminoid content in 3D-printed emulgel constructs post-exposure to visible or UV light, which was used for GelMA crosslinking, we analyzed constructs (10 × 10 × 2 mm^3^) composed of 10% (w/v) GelMA, 2% (v/v) Tween-80, and 12% (v/v) AAM-HDES containing *C. longa* extract. These constructs were exposed to either visible light (LED, 3.5 W, 550 Lm) or UV light (365 nm, 6 W) for 0 to 10 min, then homogenized in a mixture of 80% (v/v) ethanol/water : ACN (1 : 1), and centrifuged. The resulting supernatants were subjected to UV/vis spectroscopic analysis using a spectrofluorometer (FP-8200, Jasco, UK). The curcuminoid concentration was calculated against a standard curve correlating optical density at 420 nm with known curcumin concentrations. HPLC was utilized to quantify the concentrations of the three primary curcuminoids and ar-turmerone in 3D-printed emulgel constructs following exposure to either visible or UV light. After the constructs were homogenized in a solution of 80% ethanol/water : ACN (1 : 1) and centrifuged, the resulting supernatants were filtered using a 0.2 μm nylon membrane syringe filter (Agilent, USA) before HPLC analysis. This analysis was conducted under the conditions detailed in Section 2.3.

### 2.8 Mechanical testing and swilling test

3D-printed emulgel wound dressings (10 × 10 × 5 mm^3^, at least 3 samples for each experimental setup) were used to evaluate the mechanical properties and swelling behavior. Mechanical testing was conducted using a TA HD plus Texture Analyser (Stable micro systems) equipped with a 5 kg load cell in a compression mode. All the samples were compressed from top to bottom at 1 mm min^−1^ up to 50% strain, which induced deformation in the constructs, to determine Young's modulus (kPa) from the initial linear slope (5–20% strain) of the stress–strain curves. 3D printed GelMA constructs without HDES and curcuminoids extract were used as controls. To analyze swelling, the dressings were submerged in DMEM medium enriched with 70% FBS, 10 μg mL^−1^ collagen, 200 μg mL^−1^ fibrinogen, and 12 mM lactic acid, simulating the composition of wound exudate,^29^ and then incubated at 37 °C in a CO_2_ incubator. Swelling behavior was assessed by removing the dressings periodically, blotting dry, and weighing initially every 30 minutes for 2 hours, then hourly up to 8 hours, and finally at regular intervals until 192 hours. Post-incubation, samples were freeze-dried for dry weight measurement. The swelling ratio was calculated as [(swollen mass − dry mass)/dry mass] × 100%. For the stability test, each 3D-printed emulgel construct, after full crosslinking under visible light, was kept in sterile black grip-seal plastic bags at 4 °C ± 2 °C for either 7 or 28 days to simulate the scenario where the wound dressings might be stored in sterile, cold environments without exposure to light or moisture. Following storage, the samples were subjected to compression testing, swelling analysis, and curcuminoid content measurements to assess mechanical, swelling, and chemical stability over time.

### 2.9 Franz cell diffusion

Round-shaped 3D printed emulgel dressings (15 mm diameter, 5 mm thickness) containing 10% w/v GelMA, 2% v/v Tween-80, and 12% OAM-HDES containing *C. longa* extract were placed on 25 mm Strat-M® membranes in automated Franz diffusion cells (PermeGear V9-CA, USA). The receptor compartment was filled with a 10% v/v Tween-80 PBS, maintained at 37 °C and stirred at 100 rpm. PBS (1 mL) were collected from the receptor compartment at designated intervals for curcuminoid analysis *via* UV-vis spectroscopy at 420 nm absorption. Each sampling was followed by replenishing 1 mL of PBS. Curcuminoid concentrations, derived from optical absorption (OD) values against a standard curve, were used to plot the cumulative release graph.

### 2.10 Biocompatibility and proliferation of human dermal fibroblasts

Following crosslinking under visible light and a brief sterilization in DMEM with 2% (v/v) AM/AM for up to 5 minutes, 3D-printed emulgel constructs (10 × 10 × 5 mm^3^, with 200 μm inter-strand spacing) composed of 10% GelMA, 2% Tween-80 and 12% OAM-HDES with or without *C. longa* extract were seeded with 5 × 10^5^ cells of the 3rd passage of Human Dermal Fibroblasts (HDFa, C0135C, ThermoScientific) in 6-well plates. After a 3 hour adhesion period, the cultures were maintained in DMEM supplemented with 10% (v/v) FBS, 1% glutamate, 1% (v/v) non-essential amino acids, and 1% (v/v) AB/AM at 37 °C in a CO_2_ incubator, with bi-daily media changes. Samples collected on days 1, 7, 14, and 28 underwent overnight digestion at 65 °C in a Papain solution (280 μg mL^−1^ papain, 50 mM EDTA, 5 mM l-cysteine in Dulbecco's PBS pH 6.5), centrifugation at 13 000 rpm for 10 minutes, and 200-fold dilution for DNA quantification *via* the Quant-iT™ PicoGreen® assay. Initial cell viability was assessed with the LIVE/DEAD assay in DMEM containing 4 μM calcein-AM and 8 μM EthD-1 at 37 °C for 1 hour; live and dead cells fluoresced green and red, respectively. To evaluate cell adherence morphology, selected samples were freeze-dried, fixed in 2.5% glutaraldehyde/PBS and 1% osmium tetroxide/dH_2_O, sequentially dehydrated in ethanol, dried with HMDS, gold-sputtered, and then visualized by SEM (Zeiss Merlin Compact, Germany).

### 2.11 Anti-biofilm studies

To prepare a starting inoculum of approximately 10^6^ CFU mL^−1^, glass slides in a 24-well flat-bottomed plate were each filled with 990 μL TSB medium with 0.5% (w/v) glucose, then 10 μL of *S. aureus* biofilm forming strain ATCC 25923 (10^8^ CFU mL^−1^, 0.5 McFarland's standard) were inoculated to induce biofilm growth over 24 hours under static condition. Subsequently, these slides, now hosting bacterial biofilms, were positioned on 3D printed emulgel dressings (1 × 1 × 0.5 cm^3^) with 12% (v/v) OAM-HDES, including variants with *C. longa* extract, while GelMA-only dressings were used as controls. This assembly was immersed in TSB medium plus 0.5% (w/v) glucose and incubated at 37 °C for a further 7 days. Post-incubation, slides were washed twice with PBS, stained with SYTO™ 9 and propidium iodide (PI) for 15 minutes, and analyzed *via* Fluorescent confocal microscopy (Leica DMi8, Switzerland). Mean percentages of live and dead bacterial cells within the total area of the biofilm fluorescent images at 400× magnification (*n* = 3) were quantified by analyzing the intensities of green and red fluorescence using ImageJ software (NIH, USA).

### 2.12 Statistical analysis

The experimental results are presented as the mean ± standard deviation from at least three repeated experiments. Statistix 9.0 software was used for data management and analysis. The Mann–Whitney *U* test and Kruskal–Wallis One-Way ANOVA were employed to confirm statistically significant differences between two or more sample groups, respectively, at a 95% confidence level (*p* < 0.05).

## 3. Results & discussion

### 3.1 Characterization of HDES

**Fig. 1:**
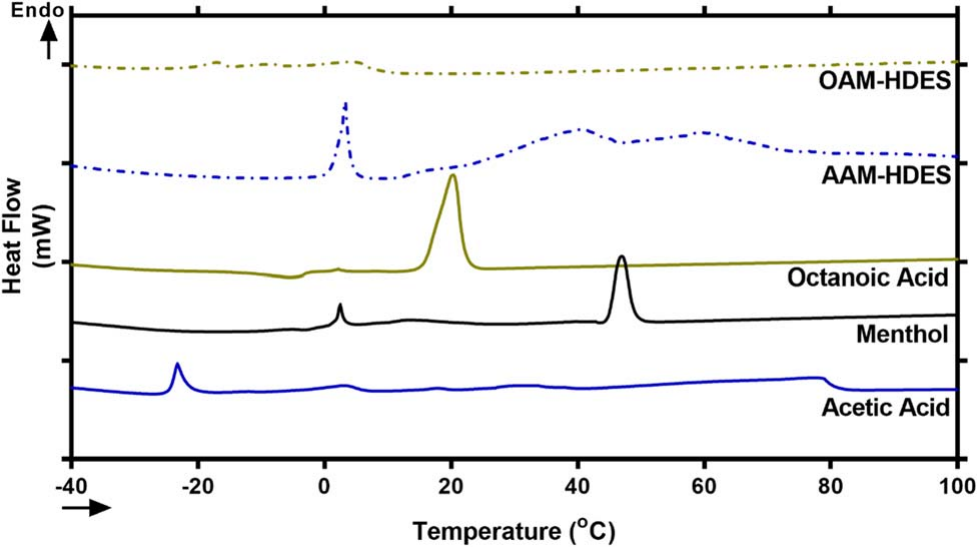
Representative DSC thermograms of individual components of HDES including acetic acid, octanoic acid, and menthol as well as the two HDES systems, AAM- and OAM-HDESs. Peaks rising above the baseline represent endothermic events.

Eutectic solvents form when two or more substances, which usually solidify at different temperatures, combine to solidify at a lower, unified temperature. This process creates a mixture with a melting point lower than any of the individual substances involved.^30^ To confirm typical characteristics of eutectic mixtures, we used DSC to analyze mixtures of acetic acid, octanoic acid, and menthol, focusing on AAM- and OAM-HDESs. The DSC thermograms (Fig. 1) showed that these mixtures melted at lower temperatures than their individual components, indicating they had formed eutectic mixtures.^30^ This behavior suggested that the acids and menthol interact to create a new phase with different thermal properties, confirmed by changes in the DSC thermograms' melting points and peak shapes.^31^ It is well established that HDES components interact through weak intermolecular forces, such as hydrogen bonds, van der Waals forces, and dispersive interactions.^32^ Specifically, a previous study utilizing mass spectrometry and molecular modeling reported that l-menthol and acetic acid in a DES system interact *via* hydrogen bonding.^33^ Another study demonstrated that dl-menthol and octanoic acid form a network of hydrogen bonds and van der Waals interactions, with longer-chain fatty acids (C8 to C12) contributing to greater hydrophobicity, enhanced aqueous stability, and stronger resistance to water penetration in HDES.^34^

While the precise mechanisms by which HDES enhances the solubility of curcuminoids and ar-turmerone remain unclear, a previous study using HDES to extract phenolic compounds from coal tar suggested that hydrogen bonding between HDES and phenolic compounds plays a key role in the extraction process.^35^ This suggests that similar hydrogen bonding interactions may facilitate the solubilization of curcuminoids and ar-turmerone in our study. However, these interactions require further investigation to be fully understood.

The lower melting points and altered peak shapes in the binary mixtures observed through DSC thermograms (Fig. 1) are classic signs of eutectic behavior, hinting at the formation of a new, more stable phase. This is consistent with previous research that has shown how components in a eutectic mixture interact to lower the overall melting point, making these mixtures valuable for applications requiring specific melting temperatures.^31^

### 3.2 Solubility of curcuminoids and ar-turmerone

The solubility of curcuminoid derivatives, including curcumin, demethoxycurcumin, and bisdemethoxycurcumin, as well as ar-turmerone, was evaluated using HDES and their individual components to extract bioactive compounds from *C. longa* rhizome ethanolic extract. These results were compared to the use of conventional solvents (*i.e.*, 95% (v/v) ethanol and distilled water) using HPLC methods (Table 1). For AAM-HDES system, which were formulated with acetic acid and menthol in a molar ratio of 2 : 1, the solubility values obtained were 7.452 ± 0.015 mg mL^−1^ for bisdemethoxycurcumin, 6.452 ± 0.038 mg mL^−1^ for demethoxycurcumin, 8.816 ± 0.027 mg mL^−1^ for curcumin, and 10.82 ± 0.04 mg mL^−1^ for ar-turmerone. In comparison, OAM-HDES system, formulated with octanoic acid and menthol in the same molar ratio, showed solubility values of 3.419 ± 0.004 mg mL^−1^ for bisdemethoxycurcumin, 2.436 ± 0.006 mg mL^−1^ for demethoxycurcumin, 1.811 ± 0.003 mg mL^−1^ for curcumin, and 5.140 ± 0.042 mg mL^−1^ for ar-turmerone.

**Table 1:** Solubility of each curcuminoid derivatives (curcumin, demethoxycurcumin, and bisdemethoxycurcumin) and ar-turmerone in HDES solutions and their individual components compared to the conventional solvents (95% (v/v) ethanol/water and distilled water)

HDES & component	Solubility (mg mL^−1^)			
	BIS^c^	DEM^c^	CUR^c^	Ar-Tu^c^
AAM-HDES^a^	7.452 ± 0.015	6.452 ± 0.038	8.816 ± 0.027	10.82 ± 0.04
OAM-HDES^b^	3.419 ± 0.004	2.436 ± 0.006	1.811 ± 0.003	5.140 ± 0.042
Acetic acid	9.398 ± 0.013	7.789 ± 0.046	9.649 ± 0.052	14.26 ± 0.05
Octanoic acid	3.176 ± 0.005	2.139 ± 0.007	1.311 ± 0.009	5.836 ± 0.111
Ethanol (95% v/v)	0.443 ± 0.084	0.544 ± 0.101	0.895 ± 0.077	0.016 ± 0.007
Distilled water	0.003 ± 0.0001	NA^d^	0.002 ± 0.0003	NA^d^

a AAM-HDES, HDES formulated by acetic acid: menthol in a molar ratio of 2 : 1.

b OAM-HDES, HDES formulated by octanoic acid: menthol in a molar ratio of 2 : 1.

c BIS, bisdemethoxycurcumin; DEM, demethoxycurcumin; CUR, curcumin; Ar-Tur, ar-turmerone.

d Less than limit of quantitation (LOQ) of the HPLC condition used (0.00198 mg mL^−1^).

The individual components, acetic acid and octanoic acid, displayed varying solubility for these compounds. Acetic acid showed the highest solubility values: 9.398 ± 0.013 mg mL^−1^ for bisdemethoxycurcumin, 7.789 ± 0.046 mg mL^−1^ for demethoxycurcumin, 9.649 ± 0.052 mg mL^−1^ for curcumin, and 14.26 ± 0.05 mg mL^−1^ for ar-turmerone. In comparison, octanoic acid presented lower solubility values. The observed solubility data has highlighted the impact of HDES composition on the solubility of curcuminoid derivatives and ar-turmerone. The AAM-HDES system exhibited higher solubility for these compounds compared to the OAM-HDES system, suggesting that the nature of the acid component significantly influences the solubility of these bioactive molecules. This aligns with our previous finding that the choice of HDES components plays a crucial role in determining the solubility of solutes.^24^

Compared to the conventional solvents, both HDES systems and their individual components significantly outperformed 95% (v/v) ethanol/water and distilled water in dissolving curcuminoids and ar-turmerone. Notably, ethanol showed relatively low solubility for bisdemethoxycurcumin (0.443 ± 0.084 mg mL^−1^), demethoxycurcumin (0.544 ± 0.101 mg mL^−1^), curcumin (0.895 ± 0.077 mg mL^−1^), and particularly low for ar-turmerone (0.016 ± 0.007 mg mL^−1^). Distilled water exhibited the least solubility, with values falling below the limit of quantitation (LOQ) for some compounds. These findings suggested that incorporating HDES, particularly those based on acetic acid, markedly improved the solubility of curcuminoid derivatives and ar-turmerone. This improvement can potentially enhance the bioavailability and efficacy of these bioactive molecules in various medical and industrial applications.

### 3.3 Formulation and characterization of gelatin-based emulgels

**Fig. 2:**
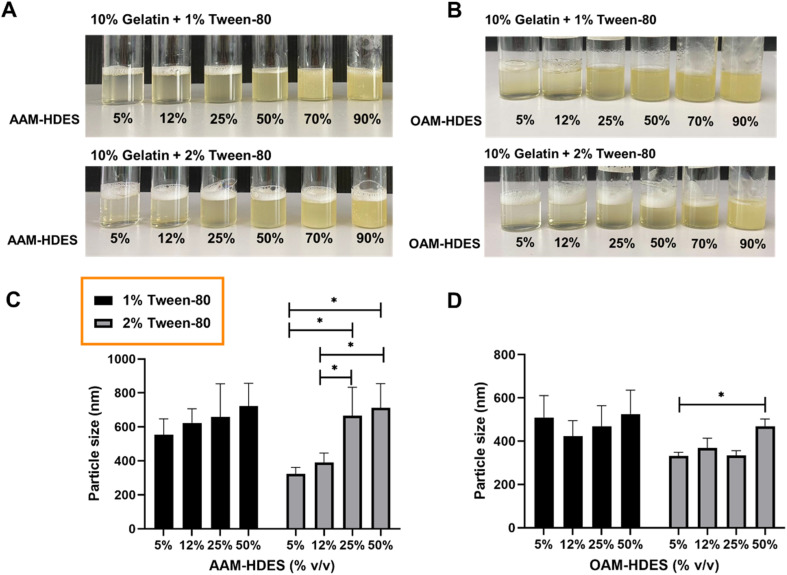
Representative images and particle size analysis of gelatin (10% w/v) emulgel formulations containing either 1% or 2% v/v Tween-80, with incremental volumes of HDES. Visual representation of gelatin solutions incorporating AAM-HDES (A), and OAM-HDES (B), demonstrating solution clarity and homogeneity at different HDES concentrations (5%, 12%, 25%, 50%, 70%, 90% v/v). DLS data indicating particle size distribution of AAM-HDES (C) and OAM-HDES (D) within the emulgel (*n* = 5, *p < 0.05).

Fig. 2 illustrated the visual and particle size analysis of gelatin-based emulgel formulations, incorporating varying volumes of HDES. These formulations contained either 1% or 2% v/v Tween-80, and demonstrate the influence of HDES concentration on solution clarity and homogeneity. The gelatin solutions with AAM-HDES (Fig. 2A) and OAM-HDES (Fig. 2B) were observed across a range of HDES concentrations (5%, 12%, 25%, 50%, 70%, 90% v/v). Clarity and homogeneity were visibly maintained up to 25% (v/v) HDES concentration, beyond which turbidity progressively increased with higher HDES content. DLS data (Fig. 2C and D) revealed a significant increase in the particle size of HDES within the emulgel formulations, indicating a direct correlation between the HDES concentration and the particle size.

The visual and particle size analyses of the gelatin emulgel formulations suggested a concentration-dependent interaction of HDES with the gelatin matrix. At lower concentrations, HDES can be uniformly dispersed within the gelatin, as evidenced by the clear gel structure. However, as the HDES concentration increases, the system's homogeneity decreases, which is caused by larger particle formation, as confirmed by the DLS data. These findings are consistent with our prior studies in the formulation of HDES microemulsion,^15^ indicating that the solute–solvent interaction becomes less favorable as the concentration of HDES increases, affecting the system's physical stability, which may have an impact on the mechanical and release properties of the formulated emulgel systems. Thus, we then selected a 12% (v/v) concentration of HDES to formulate the emulgel for subsequent 3D printing fabrication.

**Fig. 3:**
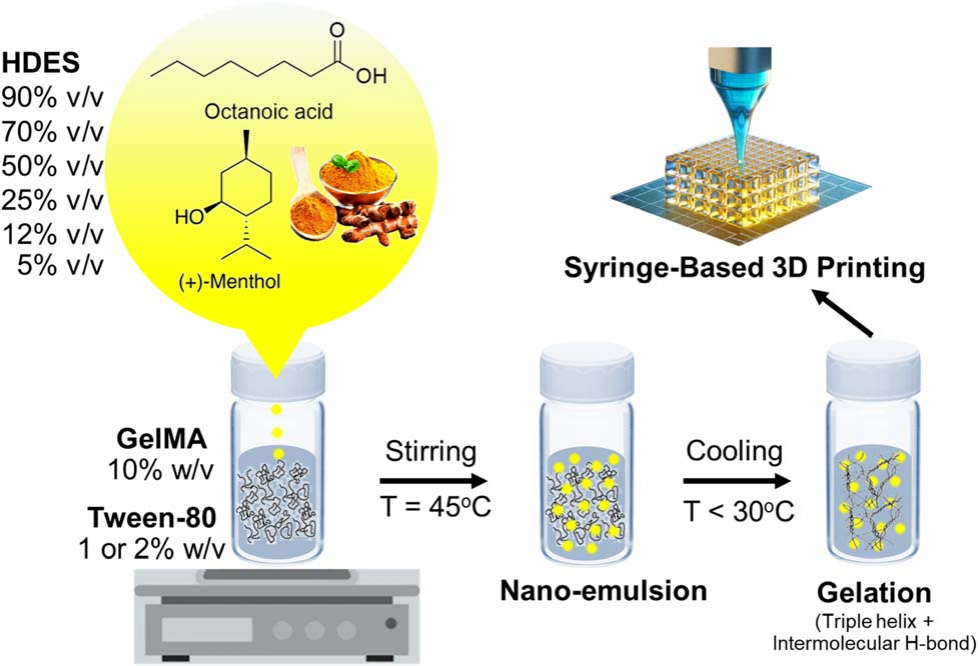
Schematic diagram illustrating the formulation of an emulgel composed of HDES containing *C. longa* extract, GelMA, and Tween-80. The resulting emulgel is suitable for use as a printing ink in the fabrication of tailored wound dressings by a custom-made syringe-based 3D printing.

The schematic in Fig. 3 hypothesized the formation mechanism of an emulgel by incorporating HDES, which acts as the oil phase, into a gelatin or GelMA solution, serving as the aqueous phase. The process entailed blending HDES with a solution of gelatin or GelMA containing Tween-80 at a temperature of 45 °C, resulting in the creation of a nanoemulsion.^36^ In this nanoemulsion, the *C. longa* extract, dispersed within the HDES, formed emulsion droplets that were stabilized by Tween-80 within the gelatin or GelMA matrix. As shown in Fig. 2, Tween-80 at concentration of 2% produced a nanoemulsion with smaller particle sizes than the 1% concentration. This suggested that higher concentrations of Tween-80 would provide more surfactant molecules to surround and stabilize individual droplets, preventing them from coalescing and resulting in smaller particle sizes. Furthermore, a denser protective layer around the HDES droplets formed by Tween-80 may reduce van der Waals attractions between them, further enhancing the stability of the nanoemulsion. For this reason, 2% Tween-80 was selected for our subsequent 3D printing experiments. It is important to note that nanoemulsions are not thermodynamically stable but are kinetically stable, meaning they can remain emulsified for extended periods due to the slow dynamics of phase separation.^29^ Surfactants like Tween-80 enhance this kinetic stability by reducing the surface tension between the oil and water phases. Upon cooling the mixture below the gelation temperature of gelatin, gelatin peptide chains changed their configuration from a random coil to a triple helix, promoting intermolecular hydrogen bonding and leading to the gelation of the solution,^37^ which could be 3D printed into a customized wound dressing.

**Fig. 4:**
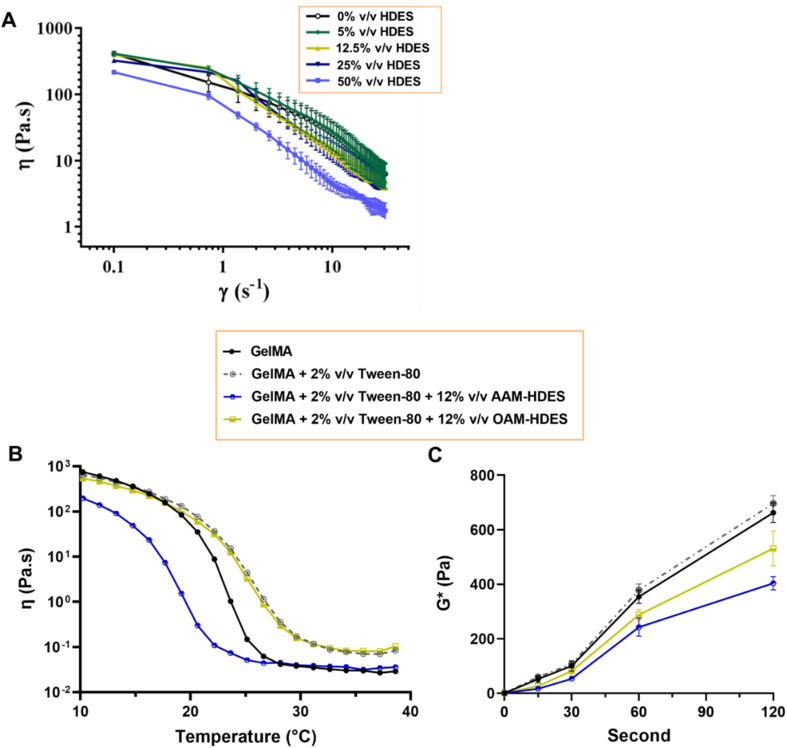
(A) Rheological properties of the emulgel using OAM-HDES showing pseudoplastic behavior. (B) Comparison of 10% GelMA gelation with and without 2% Tween-80, alongside the effect of AAM-HDES or OAM-HDES across temperatures from 10–40 °C. (C) Variation in complex modulus for gel samples under visible light exposure (60 cm distance, LED, 3.5 W, 550 Lm) over time (0–120 seconds). Data represented mean from triple measurements of three independent samples (*n* = 9).

To evaluate if the addition of HDES may potentially alter the mechanical strength and elasticity of the final gel, rheological studies were performed, providing insights into how HDES modulates the structural characteristics of gelatin and GelMA gels. The rheological behavior depicted in Fig. 4A delineated the rheological properties of emulgels formulated with OAM-HDES. The flow curves exhibited shear-thinning or pseudoplastic behavior, with viscosity (*η*) decreasing as the shear rate (*γ*) increases across all HDES concentrations tested (0%, 5%, 12.5%, 25%, 50% v/v HDES). Incorporating increasing amounts of HDES into the emulgel systematically reduced its zero-shear-rate viscosity. This reduction was more pronounced with higher concentrations of HDES, suggesting that the addition of HDES to the emulgel formulations appeared to disrupt the gel network: significantly impacting on the viscoelastic properties of the gel. In Fig. 4B, the gelation temperature profiles of GelMA gels were distinctly influenced by the addition of HDES and Tween-80. Specifically, the inclusion of OAM-HDES in the formulation led to a higher gelation temperature, while AAM-HDES lowered the gelation threshold, implying that the latter may hinder 3D printing process at ambient temperatures. The shift in gelation temperature for the AAM-HDES suggested possible denaturation or charge alteration of the gelatin peptides. It is hypothesized that the HDES containing acetic acid may interact with gelatin peptides, potentially through mechanisms such as hydrogen bonding or electrostatic interactions, leading to earlier gelation at lower temperatures.^38^ This contrasts with OAM-HDES system, which necessitates higher temperatures to induce gelation, possibly due to a less pronounced interaction with GelMA or a stabilizing effect of Tween-80 in the system. In light of this discovery, we opted to use emulgel containing OAM-HDES for subsequent 3D printing, even though it demonstrated lower solubility for curcuminoids and ar-turmerone relative to AAM-HDES. This choice was made to circumvent the issue of structural melting observed during the printing and crosslinking processes with the latter formulation. For printing emulgels derived from AAM-HDES herein, incorporating a cooling system during printing and chilling the constructs on ice during visible light crosslinking were necessary steps. Fig. 4C illustrates that visible light exposure effectively crosslinked emulgels containing 0.5% LAP photoinitiator, with an appropriate crosslinking time of around 60 seconds. This duration was sufficient for the viscosity values to stabilize, indicating complete crosslinking. This finding has aligned with previous studies demonstrating that visible light, in conjunction with LAP, provided a viable method for achieving rapid and efficient photocrosslinking of GelMA-based hydrogels containing bioactive substances.^39^

### 3.4 Photostability of curcuminoids in emulgel constructs under visible light or UV irradiation

**Fig. 5:**
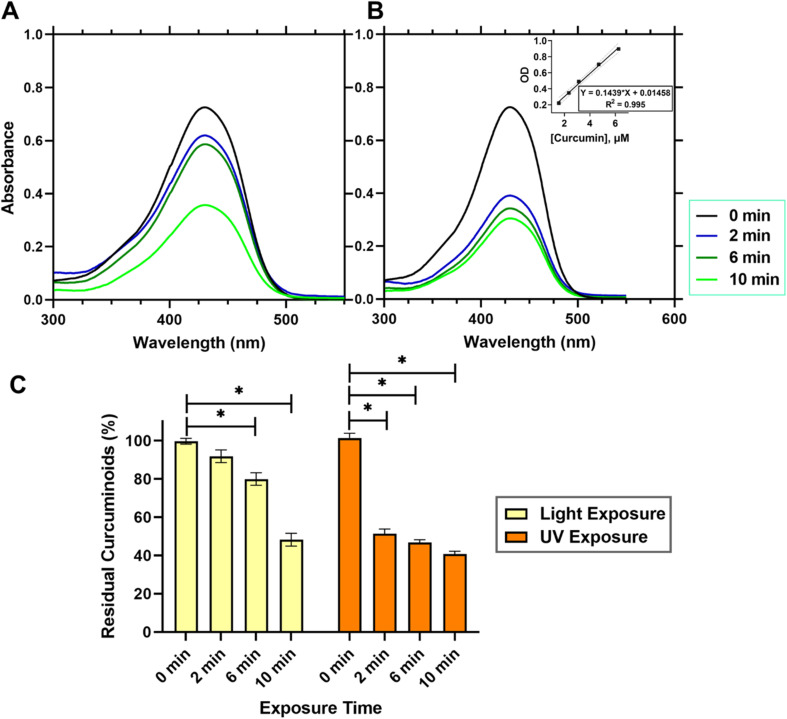
Quantitative assessment of curcuminoid content in 3D printed emulgel (10 × 10 × 2 mm^3^) with 10% gelatin and 2% Tween-80, incorporating 12% AAM-HDES with *C. longa* extract. UV absorption spectra of curcuminoids post-exposure to visible (A) and UV light (B) for GelMA crosslinking over time (0–10 min). (C) Curcuminoid quantification is derived from the standard curve shown in the inset of (B). Data are averages from nine samples (*p < 0.05).

As GelMA required exposure to visible light or UV light to initiate covalent crosslinking of methacrylic groups rendering the gel mechanically robust and stable, the photo-crosslinking using high intensity of light may cause photo-degradation of the curcuminoids loaded within the gel constructs. Fig. 5 presented the quantitative analysis of curcuminoids within a 3D printed emulgel structure in a dimension of 10 × 10 × 2 mm^3^, containing 10% gelatin and 2% Tween-80, and incorporating 12% AAM-HDES with *C. longa* extract. The UV absorption spectra (Fig. 5A and B) compared the stability of curcuminoids after exposure to visible (Fig. 5A) and UV light (Fig. 5B), respectively. Quantification of the curcuminoids (Fig. 5C) was based on a standard curve, as shown in the inset of Fig. 5B. The data indicated that after exposure to UV light, there was a significant reduction, by almost 50%, in the total curcuminoid content, whereas visible light exposure retains about 90% of the curcuminoids within the 3D printed constructs. It was clearly evident from the results that UV light exposure has a substantial detrimental effect on the curcuminoid content in 3D printed emulgel constructs. This degradation could significantly compromise the therapeutic efficacy of the wound dressing product due to the reduced bioactivity of the curcuminoids. The nearly 50% loss of curcuminoid content upon UV exposure suggested that this crosslinking method could be less favorable for formulations where bioactivity retention is critical.

In contrast, the use of visible light for crosslinking the gel post-printing demonstrated a minimal impact on the integrity of curcuminoids, retaining approximately 90% of these bioactive compounds within the gel constructs. This finding supports the potential application of visible light as a gentler crosslinking method, preserving the biological functionalities of incorporated cells, growth factors or phytochemicals.^14^

**Fig. 6:**
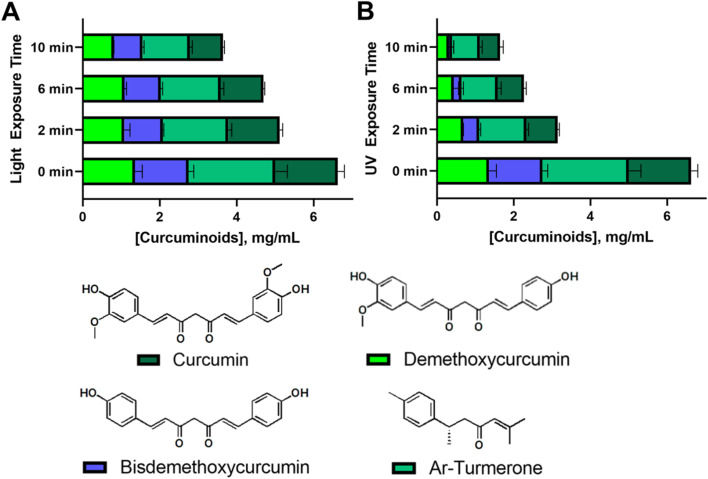
HPLC quantification of curcuminoids and ar-turmerone in 3D printed emulgel samples (10 × 10 × 2 mm^3^) containing 10% gelatin, 2% Tween-80, and 12% AAM-HDES containing *C. longa* extract. Analysis was performed post-irradiation with visible (LED, 3.5 W, 550 Lm) (A) or UV (365 nm, 6 W) light (B) at 60 cm for 0–10 min intervals (*n* = 9). Chemical structures of the analyzed compounds are depicted in the lower panels.

An extensive HPLC analysis was further conducted to assess the photostability of the four principal compounds in *C. longa* extract within consistently sized 3D printed emulgel constructs (10 × 10 × 2 mm^3^). These constructs contained 10% gelatin, 2% Tween-80, and 12% AAM-HDES with *C. longa* extract. When subjected to visible (Fig. 6A) and UV light (Fig. 6B) irradiation, a hierarchy in photostability was evident, with curcumin proving most stable under both light conditions, followed by demethoxycurcumin, bisdemethoxycurcumin, and ar-turmerone, the latter showing the most significant degradation.

Superior resistance to visible light-induced degradation demonstrated by curcumin may be attributed to its molecular architecture, including the presence of hydroxyl or methoxy groups. Demethoxycurcumin and bisdemethoxycurcumin, with fewer methoxy groups, still retained notable stability under visible light, yet were more susceptible to UV degradation. Ar-turmerone, which lacks hydroxyl groups and exhibits limited water solubility, displayed lower stability overall. However, the specific contribution of hydroxyl groups or methoxy groups to photoprotection requires further study as this finding contradicted the previous literature in which the presence or absence of these groups on phenyl ring did not have significant influence in photodegradation.^40^ This suggested that the relationship between water solubility, molecular structure, and photodegradation is complex and likely involves various factors beyond simple solubility interactions and the presence of some functional groups. Of note, the understanding of photostability is crucial for the development of 3D printed constructs for therapeutic applications, as it directly affects the bioefficacy of embedded compounds. Our results have suggested that visible light crosslinking methods may be preferable for curcuminoid-containing tissue-engineered constructs (*i.e.*, a personalized wound dressing).

### 3.5 Characterization of 3D printed emulgel constructs

**Fig. 7:**
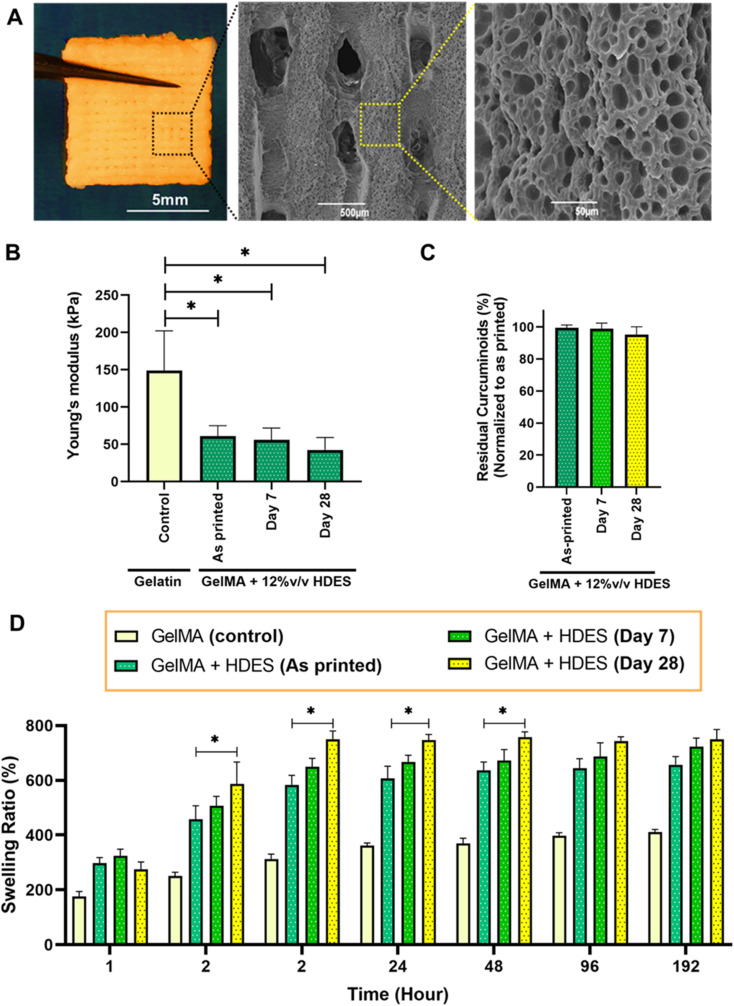
(A) Macroscale image of a fully crosslinked 3D-printed emulgel construct, formulated with 12% (v/v) OAM-HDES containing *C. longa* extract, 10% (w/v) GelMA, and 2% (v/v) Tween-80, along with representative SEM images showing surface topographies post-freeze-drying. The mechanical and chemical stability of the constructs (as-printed *vs.* constructs stored for 7 and 28 days in sterile grip-seal plastic bags without light exposure at 4 °C) was evaluated by (B) compressive moduli, (C) residual curcuminoid content, and (D) swelling ratios. Pure GelMA constructs were used as control materials (*n* = 3, *p < 0.05 compared to as-printed structures).

Fig. 7A represented a macroscale image of a 3D printed emulgel construct composed of 12% (v/v) OAM-HDES containing *C. longa* extract, 10% (w/v) GelMA and 2% (v/v) Tween-80 in a lattice structure. The emulgel formulation also included 0.5% lithium phenyl-2,4,6-trimethylbenzoylphosphinate (LAP), serving as a photoinitiator that facilitates the covalent crosslinking of methacrylic groups upon exposure to visible light,^14^ thus yielding a mechanically stable yellow opaque appearance gel construct. The lattice structure observed in the SEM images after freeze-drying revealed a highly microporous surface characteristic, which would mimic microarchitecture of skin extracellular matrix and be conducive to cell adhesion and proliferation.^41^ Compressive modulus assessment (Fig. 7B) indicated that the inclusion of HDES into GelMA matrix somewhat compromised its compressive mechanics. The observed decrease in mechanical properties upon HDES incorporation aligns with the known effect of HDES in lowering the viscosity of gelatin solutions (Fig. 4A), which could lead to a less dense crosslinked network. Swelling ratio measurements (Fig. 7C) also demonstrated an accelerated hydration rate for the constructs containing HDES. The quicker absorption of water by the HDES-containing constructs could be attributed to the decreased gel viscosity. A lower-viscosity gel matrix could lead to a looser network structure upon crosslinking, which, in turn, could accommodate water molecules more readily, resulting in a higher swelling ratio. While octanoic acid displayed relative hydrophobicity compared to gelatin, it possessed a polar carboxylic group, and menthol includes a hydroxyl group, which may likely to promote enhanced water molecule integration within the gel network. However, the intricate interactions among the formulation constituents yield a multifaceted swelling behavior, underscoring the necessity for an in-depth comprehension of the interplay within the hydrogel matrix.

By evaluating both the mechanical (Fig. 7B and D) and chemical stability (Fig. 7C) of the 3D-printed wound dressing after 7 and 28 days of storage in sterile grip-seal plastic bags without light exposure at 4 °C ± 2 °C, we aimed to emulate potential clinical scenarios where immediate bedside production of the 3D printed personalized wound dressings may not be feasible, and temporary storage is required. After 7 days of storage, both the compressive moduli (Fig. 7B) and the residual curcuminoid content (Fig. 7C) remained comparable to those of the as-printed constructs, indicating that the wound dressings retained sufficient mechanical strength and stable curcuminoid content, ensuring their suitability for clinical applications during short-term storage.

However, after 28 days of storage, the mechanical stability began to deteriorate, as evidenced by a significant increase in swelling ratio (Fig. 7D) compared to the as-printed constructs. This loss of stability could be attributed to physical aging or the hydrolysis and degradation of the hydrogel network over time, which likely weakened the crosslinked structure and affected its mechanical properties.^42^ These findings suggest the importance of optimizing storage conditions and usage timelines to ensure the effectiveness and structural integrity of 3D-printed wound dressings.

**Fig. 8:**
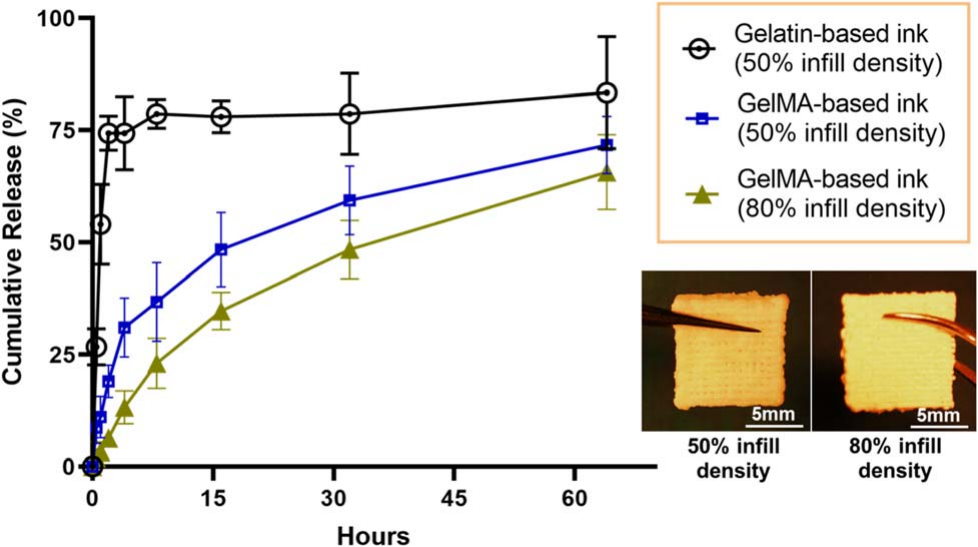
Total curcuminoid release from 3D-printed, fully crosslinked emulgel constructs (formulated with 12% OAM-HDES containing *C. longa* extract, 10% w/v GelMA, and 2% v/v Tween-80). The release was measured over 60 hours across a Strat-M® membrane using an automated Franz diffusion cell system, comparing constructs with 50% infill density and 80% infill density, as well as constructs where gelatin was used as a substitute for GelMA (*n* = 3).

The release kinetics of curcuminoids from 3D-printed emulgel constructs (Fig. 8) were evaluated over 60 hours using an automated Franz cell system with a Strat-M® membrane, which simulated the human skin barrier to assess transdermal delivery potential.^43^ Constructs containing GelMA demonstrated a more sustained release of curcuminoids compared to those with gelatin, likely due to GelMA's covalent crosslinking, which created a stable matrix that retained curcuminoids and controls their release. In contrast, gelatin constructs, which lack covalent crosslinking, exhibited a faster release, suggesting limited curcuminoid retention.

Infill density was also shown to influence release behavior. Constructs with 80% infill density exhibited a lower cumulative release compared to those with 50% infill density, indicating that the denser matrix with less internal macropores likely improved curcuminoid encapsulation, enabling a gradual, sustained release over time. In comparison, the more porous structure of the 50% infill construct allowed for faster initial diffusion but may have compromised the sustainability of release.

Our findings highlight the customization potential of 3D printing in developing wound dressings with tunable release profiles, achieved by adjusting structural parameters, for instance, infill density. Optimizing infill density has proved to be one of the effective strategies for enhancing the performance and therapeutic value of bioactive dressings. The successful permeation of curcuminoids through the Strat-M® membrane also demonstrated the applicability of these constructs for their ability to deliver active compounds across the skin barrier. In particular, the gradual release profile observed with a higher infill density ensured sustained exposure of the wound to curcuminoids, which could enhance their anti-inflammatory and antimicrobial effects. These results emphasize the importance of balancing material composition and structural design to achieve optimal release kinetics for effective 3D-printed wound dressings in clinical settings.

### 3.6 Biocompatibility of 3D printed emulgel constructs with human dermal fibroblasts

**Fig. 9:**
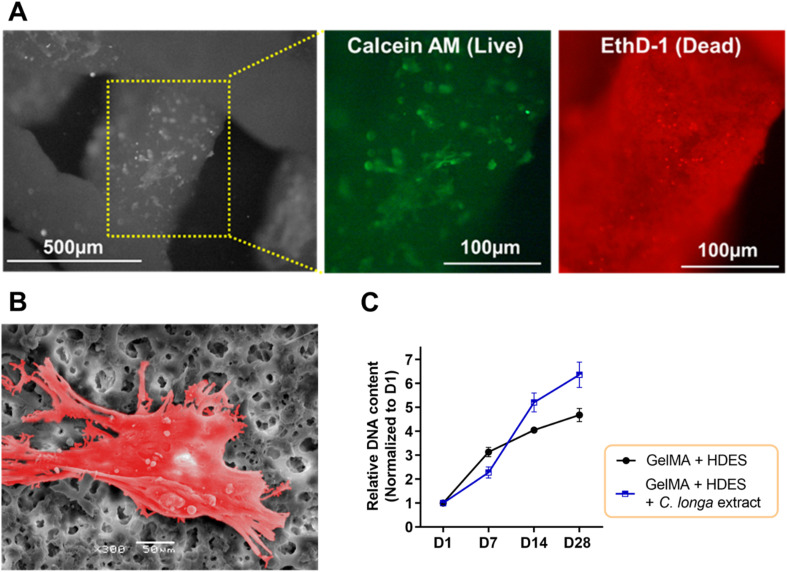
(A) Human dermal fibroblast viability on 3D printed, fully crosslinked emulgel constructs (12% OAM-HDES containing *C. longa* extract, in 10% GelMA, 2% Tween-80) assessed by live–dead assay, with live cells in green and dead cells in red. (B) SEM image of fibroblast adhesion on the emulgel, with cells highlighted in red for clarity. (C) Fibroblast proliferation in the 3D printed emulgel constructs containing only OAM-HDES alone or with *C. longa* extract, cultured in DMEM with 10% FBS, traced by an increasing DNA content over 28 days (*n* = 3).

To evaluate cell–material interaction, Fig. 9 provided a multifaceted evaluation of human dermal fibroblast interaction with 3D printed emulgel constructs. The emulgel, composed of a 10% GelMA and 2% Tween-80 matrix incorporating 12% OAM-HDES with 12% *C. longa* extract, demonstrated cell viability post-crosslinking *via* a live–dead assay. The predominance of green fluorescence (Fig. 9A) indicated a considerably higher number of live cells stained with calcein AM compared to the dead cells stained with ethidium bromide homodimer-1 (red), suggesting successful maintenance of cell viability which highlighted the constructs' biocompatibility. The representative SEM image (Fig. 9B) illustrated fibroblasts firmly adhered to the emulgel matrix, characterized by cellular spreading and the extension of protrusions, which are indicative of active exploration and interaction with their surrounding environment. The ability of cells to spread and exhibit protrusions is a behavior essential for robust cell–matrix interactions, crucial for the subsequent processes of tissue integration and regeneration.^44^

Proliferation assays revealed an upward trajectory of DNA content over 28 days (Fig. 9C), with a notable increase in fibroblast number on constructs loaded with *C. longa* extract, pointing to enhanced cellular growth when compared to constructs lacking the extract. The curcuminoids and other bioactive molecules within *C. longa* extract are known to promote cell proliferation and could impart additional therapeutic effects, such as anti-inflammatory responses, which are beneficial for wound healing applications.^15,17^ Constructs without the extract demonstrated less pronounced cell growth, indicating that the presence of *C. longa* extract potentially offers the chemical cues that are more conducive to fibroblast proliferation.^17^

Curcumin is well known to play a critical role in multiple mechanisms involved in wound healing. It not only eradicates free radicals and suppresses inflammation, both of which can impair the healing process, but also promotes collagen deposition, keratinocyte migration, and granulation tissue formation, accelerating wound contraction.^45,46^ Our 3D-printed wound dressing contains *C. longa* extract, which comprises four curcuminoid derivatives and ar-turmerone. However, it remains unclear whether the observed bioactivity—such as the promotion of fibroblast cell proliferation—originates from individual compounds or from the synergistic action of all components. Given that curcumin is present in the highest concentration (Fig. 6), it is likely that it plays a dominant role in the bioactivity of the extract. However, previous studies have also highlighted the importance of curcuminoid derivatives and ar-turmerone in wound healing through their anti-inflammatory effects. Specifically, the methoxy groups on the phenyl ring of curcumin derivatives significantly influence their activity. Curcumin, with two methoxy groups, exhibits the highest inhibitory potency toward TNF-induced NF-κB activation, followed by demethoxycurcumin (one methoxy group) and bisdemethoxycurcumin (no methoxy group)^47^ By contrast, ar-turmerone, which lacks methoxy groups, does not inhibit NF-κB activation. This diverse range of activities makes curcuminoids promising agents for 3D-printed wound dressings, where their combined effects may improve healing outcomes. Further studies are required to fully explore the individual and synergistic roles of these curcuminoids and ar-turmerone in wound healing.

### 3.7 Anti-biofilm activity of 3D printed emulgel constructs

**Fig. 10:**
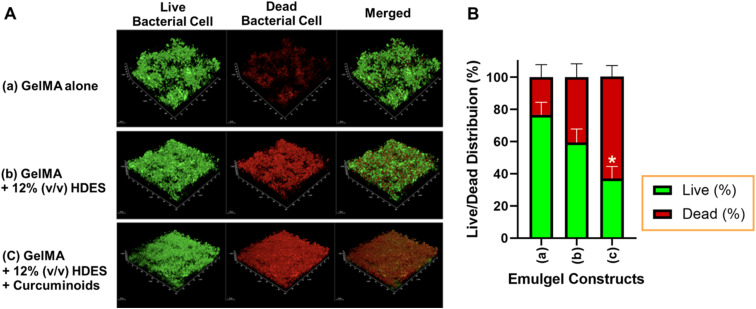
(A) Representative images of biofilm inhibition by 3D printed emulgel constructs against *S aureus*. Constructs include (a) 10% GelMA alone, (b) 10% GelMA with 12% OAM-HDES, (c) 10% GelMA with 12% OAM-HDES containing *C. longa* extract. (B) Semi-quantification of the live/dead bacterial cells in the biofilm images. The bar graph shows the mean percentage of live (green) and dead (red) bacterial cells for each construct. Error bars represent the standard deviation (*n* = 3). * indicates statistically significant differences compared to the control (10% GelMA alone, *p* < 0.05).

The effectiveness of 3D printed personalized wound dressings for chronic wounds, especially in infection control, relies on their anti-biofilm, antibacterial, and antifungal properties. In this study, the biofilm inhibitory capabilities of various 3D printed emulgel constructs against *S. aureus* ATCC25923 were assessed (Fig. 10). Constructs were based on a 10% GelMA formulation, with variations incorporating OAM-HDES and *C. longa* extract within the HDES. The live/dead assay revealed that the constructs 3D printed from ink containing GelMA alone was less effective in inhibiting biofilm formation, as indicated by the prevalent green fluorescence, with 77.6 ± 6.6% of bacterial cells still alive (Fig. 10B). The addition of OAM-HDES enhanced biofilm inhibition, as evidenced by a decreased percentage of live bacterial cells (Fig. 10B). The construct containing *C. longa* extract exhibited the most significant biofilm inhibition, with a substantial presence of red fluorescence (Fig. 10A) and a statistically significant decrease in the percentage of live bacterial cells to 36.8 ± 8.7% compared to GelMA alone (Fig. 10B). These findings suggest that the antimicrobial efficacy of GelMA constructs was markedly improved with the incorporation of OAM-HDES and further augmented by *C. longa* extract.

The integration of *C. longa* extract, renowned for the antimicrobial activity of its curcuminoids, offers a potent combination with HDES in combating bacterial biofilm formation. Curcuminoids are known to disrupt cell membranes and interfere with biofilm development, making the combination particularly effective.^48,49^ The enhanced biofilm resistance of these materials is critical in preventing wound infections and is particularly pertinent for the development of antimicrobial coatings for medical devices and advanced wound care solutions.^50^ The employment of natural bioactive compounds within 3D printed constructs presents a promising strategy for inhibiting bacterial colonization and biofilm formation, crucial for tissue engineering and regenerative medicine applications.

Chronic wounds, such as venous leg ulcers, diabetic foot ulcers, and pressure ulcers, are frequently colonized by *S. aureus* and *Pseudomonas aeruginosa*, two of the most common pathogens in non-healing wounds.^51^ These bacteria are particularly problematic because they tend to form biofilms, which protect them from antibiotics and immune responses, prolonging the healing process and making infections harder to treat. While curcumin is recognized for its broad-spectrum antibacterial activity against both Gram-positive and Gram-negative bacteria, some studies have shown that it is more effective against Gram-positive bacteria, such as *S. aureus*, due to its absence of an outer membrane that otherwise limits curcumin's penetration into Gram-negative bacteria.^52^ Additionally, menthol-based HDES have demonstrated strong antimicrobial effects, particularly against *S. aureus* and *Candida albicans*, and have shown promising activity against *P. aeruginosa.*^53^ However, further research is required to fully explore the antimicrobial potential of these 3D-printed wound dressings against Gram-negative bacteria and fungal pathogens to optimize their clinical applications.

Indeed, this work successfully extracted curcuminoids and ar-turmerone using eco-friendly HDES and incorporated them into a novel emulgel system suitable for 3D printing personalized wound dressings. Curcuminoid photostability followed a clear hierarchy, with curcumin being the most resilient due to its methoxy groups. Visible light crosslinking was identified as a superior strategy to minimize the degradation of these bioactive compounds. Further investigation into enhancing long-term UV stability and *in vivo* efficacy is warranted. This research lays the foundation for developing advanced wound dressings combining the therapeutic benefits of natural extracts with the customization enabled by 3D printing technologies.

## 4. Conclusion

Our work presents a novel application of HDES for 3D printing of a personalized wound dressing, harnessing the eco-friendliness and solubilizing power of HDES to extract and incorporate curcuminoids and ar-turmerone from *Curcuma longa* into GelMA matrices. The formulated emulgel, characterized by an optimized rheological profile, demonstrates not only robust mechanical stability but also a controlled swelling behavior, tailored for 3D printing applications. The viscoelastic properties of the gel, crucial for maintaining shape fidelity during and after printing, could be fine-tuned through the incorporation of HDES, which modulates the gel's crosslink density. The HDES system, particularly the menthol component, works synergistically with curcuminoids to bolster the anti-biofilm properties of the GelMA hydrogel. This synergy is evidenced by the marked biofilm inhibition against *Staphylococcus aureus*, offering a promising strategy for the prevention of wound infections. Furthermore, the controlled release profile of curcuminoids from the emulgel aligns with the therapeutic requirements for chronic wound management, where sustained delivery can support the prolonged healing process. Altogether, the outcomes of this work highlight the originality of using HDES to refine the functional attributes of photo-crosslinkable hydrogels. The ability to fine-tune the release kinetics while ensuring bioactivity and structural integrity post-printing exemplifies the potential of this approach to contribute to the design of advanced wound dressings for chronic wounds.

## Data availability

The raw and processed data required to reproduce these findings are part of an ongoing study and cannot be shared at this time. However, detailed descriptions and summaries of the data have already been included within the article. Further inquiries can be directed to the corresponding author.

## Author contributions

GY: conceptualization, methodology, investigation, formal analysis, writing – original draft. JJ: methodology, investigation, formal analysis, writing – original draft. RGB: writing – reviewing and editing. AP: conceptualization, methodology, investigation, formal analysis, visualization, writing – original draft, writing – review & editing, project administration, funding acquisition. All authors approved the manuscript version to be published.

## Conflicts of interest

This work declared no conflict of interest.
